# Effects of Different Adduct Ions, Ionization Temperatures, and Solvents on the Ion Mobility of Glycans

**DOI:** 10.3390/molecules30102177

**Published:** 2025-05-15

**Authors:** Hao Feng, Takumi Yamaguchi

**Affiliations:** 1 School of Materials Science, Japan Advanced Institute of Science and Technology, 1-1 Asahidai, Nomi 9231292, Japan; 2 Graduate School of Pharmaceutical Sciences, Nagoya City University, 3-1 Tanabe-dori, Mizuho-ku, Nagoya 4678603, Japan; 3 Exploratory Research Center on Life and Living Systems (ExCELLS), National Institutes of Natural Sciences, 5-1 Higashiyama, Myodaiji, Okazaki 4448787, Japan

**Keywords:** glycans, conformational dynamics, ion mobility spectrometry, mass spectrometry, electrospray ionization

## Abstract

The structural analysis of glycans remains a major challenge due to their high isomeric complexity and conformational flexibility arising from diverse glycosidic linkages and dynamic three-dimensional structures. Ion mobility–mass spectrometry (IM–MS) has been attracting attention as a way to develop the structural analysis of glycans. In this study, the effects of ionization conditions—including different types of adduct ions, ionization temperatures, and solvent environments—on the ion mobility behavior of glycans were systematically investigated. IM–MS measurements of ethylamine-tagged glycans showed broad arrival time distributions of monoprotonated ions indicating the presence of multiple conformers of glycans. Increased ionization temperatures and the use of methanol as a solvent further broadened the distribution, suggesting the enhanced conformational dynamics of the glycan ions. In contrast, sodium adduct ions yielded narrower distributions, implying that the interactions between sodium ions and glycans constrained structural flexibility. These results demonstrate that ionization parameters have a significant impact on glycan conformational behavior and mobility in the gas phase. This study provides insights into the analytical conditions for IM–MS measurements of glycans and highlights the utility of this method as a powerful tool for elucidating glycan structure and dynamics.

## 1. Introduction

Glycans, which are mainly found in glycoconjugates such as glycoproteins and glycolipids, are involved in various biological phenomena such as cellular recognition and viral infections [1,2]. Investigating the structures of glycans is important to understand the mechanism underlying their biological functions [3]. However, glycans usually have complex primary structures because of the variety of glycosidic bond patterns and highly dynamic three-dimensional structures because of the flexibility of each glycosidic bond [4]. Therefore, experimental methods for structural analysis of glycans remain limited.

Mass spectrometry (MS) and nuclear magnetic resonance (NMR) spectroscopy are commonly used for structural analysis of glycans [5]. The tandem mass method is a common technique for obtaining information on the primary structure of glycans, but MS can hardly distinguish glycan isomers because of different residue sequences and linkage types [5,6]. NMR is a potentially powerful method that can analyze not only the primary structure but also the three-dimensional structure of glycans [7,8,9]. However, considering that the timescale of the conformational dynamics of glycans is generally faster than that of NMR spectroscopy, the three-dimensional structural information of glycans obtained by NMR becomes the result of averaging the data of multiple conformers. Moreover, experimentally evaluating the distribution of multiple glycan conformations remains difficult. Thus, analyses combining computational approaches are preferably used [10,11].

Ion mobility analysis combined with MS has been attracting attention as a way to develop the primary structure analysis of glycans [5,12,13]. In ion mobility–MS (IM–MS) measurements, an ion mobility cell introduced between the ionization source and the mass analyzer allows the separation of sample ions based on their mobility in accordance with charge, size, and shape [14,15]. The collision cross-section value is estimated on the basis of the drift time data of the sample ions obtained from the IM–MS measurements, which is useful in distinguishing glycan isomers [16,17,18]. At present, a database of collision cross-section values of glycans and high-throughput analytical methods combining liquid chromatography and IM–MS techniques are being developed for primary structural analysis of glycans [19,20].

IM–MS methods also contribute to structural biology, including the analysis of the three-dimensional structure of biomacromolecules such as proteins and protein complexes [15,21]. For example, the distribution of the conformational state of dynamic protein complexes was studied by using IM–MS measurements in conjunction with soft ionization techniques such as the native MS method using electrospray ionization (ESI) [22]. The conformational characterization of N-glycans, which is a major class of glycoprotein glycans, in the gas phase was also performed using IM–MS and molecular simulations [23,24]. The IM–MS approach can provide information on the distribution of glycan conformers. However, the conditions suitable for mobility analysis of glycan ions remain to be explored. Here, the effects of different types of adduct ions, as well as different ionization temperatures and solvents, on the results of the mobility analysis of glycan ions were investigated.

## 2. Results

In glycan MS measurements, samples ionized as proton or sodium adduct ions or by deprotonation are often analyzed [17,25]. The site of protonation/deprotonation or sodium ion binding may affect the three-dimensional structure of the ionized samples; however, these positions in glycan ionization are generally ambiguous. This limitation could be unfavorable for mobility analysis, which considers the shape of the observed ions. Glycans modified by 2-aminopyridine, which is a typical fluorescent label, have also been used for IM–MS analysis [19]. The pyridine unit produces protonation, but the pyridylaminate derivatives of glycans contain an open-chain form at the reducing end.

**Figure 1:**
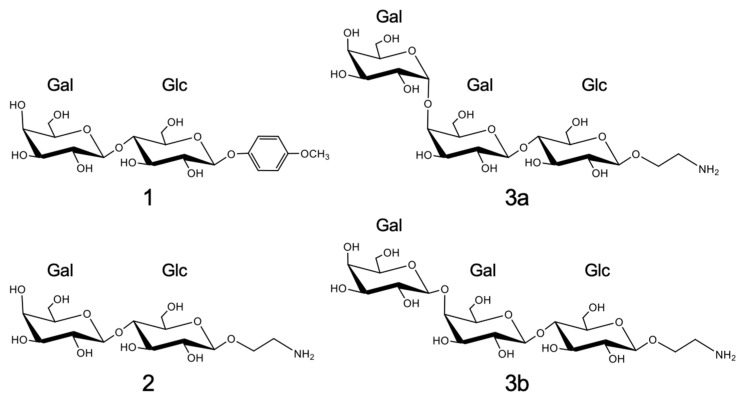
Representations of the glycans.

**Scheme 1:**
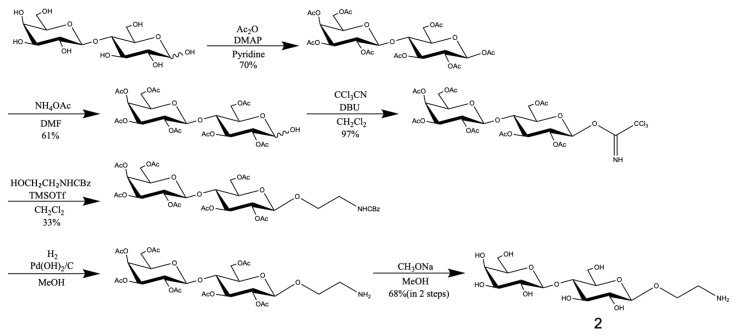
The preparation of the Galβ1-4Glcβ disaccharide, glycan **2**. Commercially available D-(+)-lactose was converted to a glycan donor, hepta-o-acetyl-lactose-1-o-trichloroacetimidate, in three steps. The glycan donor reacted with 2-(benzyloxycarbonylamino)-1-ethanol, followed by a two-step deprotection reaction to obtain glycan **2**. High-resolution MS: *m*/*z* calcd for C_14_H_28_NO_11_: 386.16569 [M+H]^+^; found: 386.16554.

**Figure 2:**
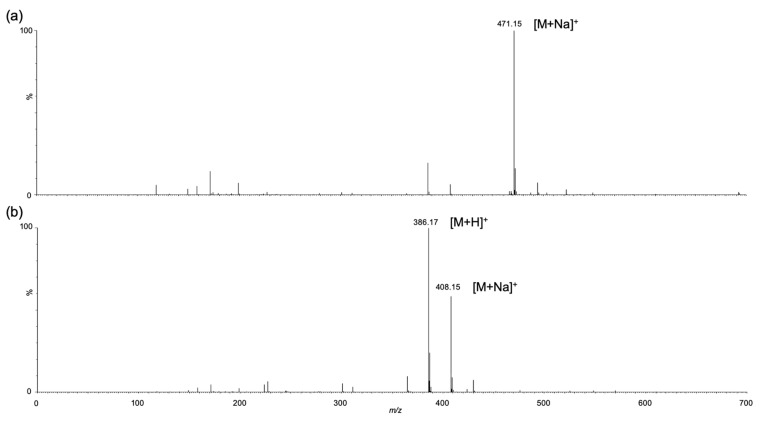
Mass spectra of Galβ1-4Glcβ disaccharides (**a**) **1** and (**b**) **2**, which contain a methoxyphenyl group and an aminoethyl group, respectively.

In generating monoprotonated ions suitable for characterizing glycan structures, an ethylamine tag was attached at the reducing end of the glycans (Figure 1). A disaccharide with the ethylamine tag (**2**) was prepared in this study, which contained galactose (Gal) and glucose (Glc) residues (Scheme 1). The sample was dissolved in water, and its ESI–MS spectrum was compared with that of Galβ1-4Glcβ disaccharide **1**, which has a methoxyphenyl group at the reducing end. In the mass spectrum of disaccharide **1**, its sodium adduct ion was observed dominantly (Figure 2a). On the contrary, the introduction of the ethylamine tag clearly produced a monoprotonated ion in addition to the sodium adduct ion (Figure 2b). The results indicated that the introduction of the tag caused the protonation of the sample to occur preferentially in the aminoethyl group rather than in the glycan body. Therefore, in this study, we aimed to observe the mobility of the induced monoprotonated ion by IM–MS measurements, which would reflect the shape of the glycans more clearly.

**Figure 3:**
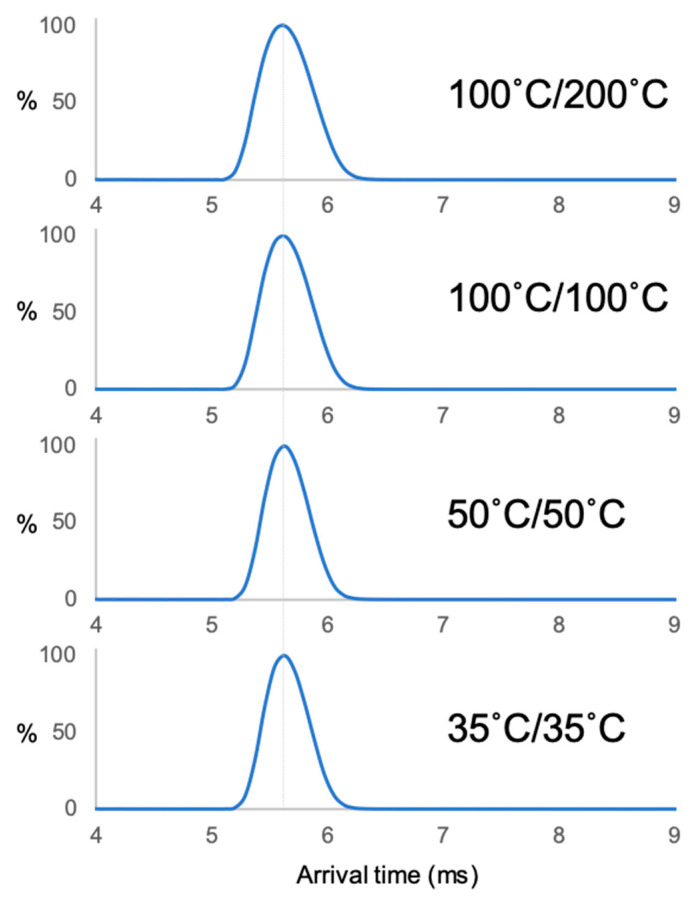
The arrival time distribution of the proton adduct ion of glycan **2** for varying ion source temperatures/desolvation gas temperatures. The dotted line indicates the estimated arrival time (5.64 ms) at 35 °C/35 °C.

Ion mobility analysis of glycan **2** was performed using an ESI–IM–MS system equipped with a traveling wave separator. The resulting proton adduct ion was selected using a quadrupole mass filter, and the mobility data were obtained. ESI conditions such as the capillary temperature influence the internal energy distribution of provided ions [26]. In traveling wave ion mobility spectrometry, the ion mobility depends on an effective temperature of the drift region, taking into account the drift gas temperature and the electric field contribution [27,28]. In studying the effect of ionization temperatures on the arrival time distribution of glycan ions, the measurements were conducted by varying the ion source temperature and desolvation gas temperature during ESI under constant mobility analysis conditions (Figure 3). The result showed that the arrival time of the glycan ion at each temperature was almost identical, but the line shape of the arrival time distribution was broader at higher temperatures than at lower temperatures. After performing three measurements at each temperature, the average range of the arrival time at half of the maximum intensity was approximately 0.43 (SD:0.001) ms at 35 °C/35 °C and 0.52 (SD:0.001) ms at 100 °C/200 °C for the ion source temperature/desolvation gas temperature. In addition, the overall line shape of the arrival time distribution observed at higher temperatures was non-normal, indicating the coexistence of multiple conformations of the glycan ions. These observations suggest that the line shape in the drift time data of the obtained glycan ions could reflect the flexibility of the structure of the glycans.

The differences in the behavior of the proton and sodium adduct ions of the glycans in mobility analysis were also investigated. Two isomeric trisaccharides with the ethylamine tags Galα1-4Galβ1-4Glcβ (**3a**) and Galβ1-4Galβ1-4Glcβ (**3b**), which differ in the α/β type of the Gal-Gal glycosidic linkage, were prepared to compare the drift time data (Figure 1). The Galα1-4Galβ1-4Glcβ trisaccharide is a glycan moiety of the glycosphingolipid Gb3 [29]. Both trisaccharides were synthesized from a Galβ1-4Glcβ disaccharide derivative (Schemes S1 and S2).

**Figure 4:**
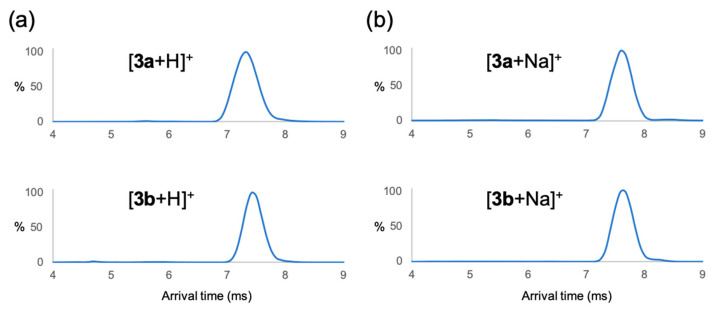
Arrival time distribution of (**a**) proton and (**b**) sodium adduct ions of glycan **3a** and glycan **3b**.

Using the ESI–IM–MS system, the proton adduct ions of these two glycans were analyzed (Figure 4). The arrival time of the glycan **3a** ion (7.32 (SD:0.001) ms) was smaller than that of the glycan **3b** ion (7.45 (SD:0.001) ms), and the overall line shape of the arrival time distribution was wider than that of the glycan **3b** ion (0.46 (SD:0.005) ms and 0.38 (SD:0.001) ms, respectively). This result indicated that the Galα1-4Gal linkage is more flexible than the Galβ1-4Gal linkage, and the Galα1-4Galβ1-4Glcβ glycan can easily conform with smaller collision cross-sections. On the contrary, mobility analysis of the sodium adduct ions showed that the arrival times of the glycan **3a** and **3b** ions were almost identical (7.61 (SD:0.001) ms and 7.63 (SD:0.001) ms, respectively). The range of the arrival time distribution of the sodium adduct ion of **3a** (0.40 (SD:0.002) ms) was smaller than that of the proton adduct ion of **3a**, indicating that the interaction with the sodium ion could perturb the conformational dynamics of the glycan. 

**Figure 5:**
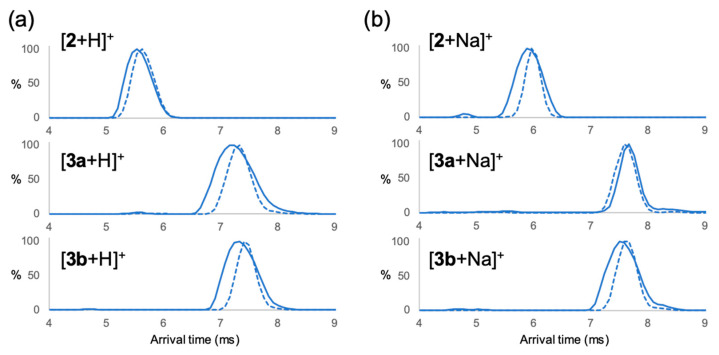
The arrival time distribution of (**a**) the proton and (**b**) sodium adduct ions of glycans **2**, **3a**, and **3b** with methanol (solid line) and water (dotted line).

**Figure 6:**
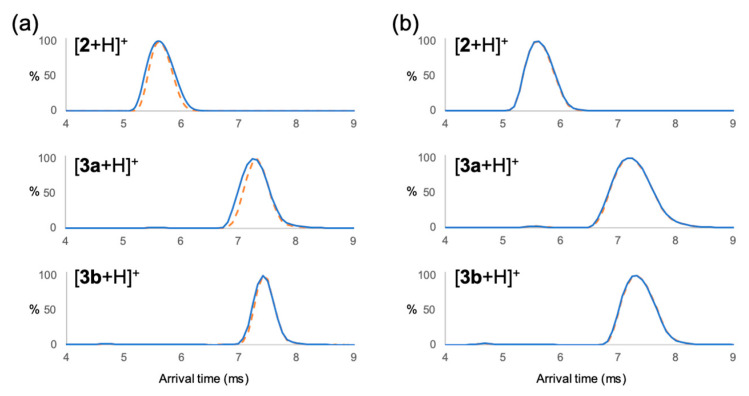
The arrival time distribution of the proton adduct ions of glycans **2**, **3a,** and **3b** (**a**) with water and (**b**) with methanol at 35 °C/35 °C (dotted line) and at 100 °C/200 °C (solid line) for the ion source temperature/desolvation gas temperature.

Previous reports indicate that the solvent for ESI can influence the drift time data [30,31,32]. For example, Chung et al. reported that the ion mobility-derived collision cross-sectional values of small molecules vary with the solvent composition [31]. In investigating the possibility that the solvent affects the drift time data of glycan ions, ion mobility measurements were performed using methanol. The solvent for the ESI–IM–MS system was replaced with methanol, and each ethylamine-tagged glycan dissolved in methanol was injected. The arrival time distributions of the glycans observed with methanol were significantly different from those observed with water. The arrival time ranges of the proton adduct ions of each glycan were larger with methanol than they were with water (Figure 5a). This difference may have been mainly due to the difference in the desolvation energy of the different solvents. Considering that methanol requires less desolvation energy than water, the ions would have more internal energy that can be converted to molecular motion [33], resulting in greater conformational dynamics in the glycans. The effects of ionization temperatures and solvents were also investigated (Figure 6). The results showed that the change in arrival time distribution at different ionization temperatures was smaller with methanol than with water. These differences in response to the ionization temperatures were presumably due to differences in the degree of dissociation of the solvent clusters [31]. 

The arrival time ranges of the sodium adduct ions of glycan **2** and glycan **3b** were also larger with methanol than they were with water (Figure 5b). For the sodium adduct ion of glycan **3a**, the arrival time changed slightly, but the ranges were almost identical with water and with methanol. The results suggest an efficient interaction between glycan **3a** and sodium ions. Meanwhile, although the IM–MS method is a measurement performed in the gas phase, the drift time data could be affected not only by the ionization conditions but also by the sample solution conditions. The contribution of solvents to protein conformers in ion mobility analysis has been studied. For example, the proportion of gas-phase ubiquitin ion conformers, such as folding and unfolding conformers, is sensitive to the ionization temperature and solvent composition [32]. For glycans, it has been reported that NMR and molecular simulation analyses suggest solvent-dependent conformational changes in disaccharides [34]. Therefore, further studies on the relationship between the conformational changes in glycans in solution and ion mobility in different solvents will be necessary. Also, note that because the traveling wave system which generally operates at high fields was used in this study [35], other types of instrumentation, such as drift tube ion mobility, may yield different observations [15,21].

## 3. Materials and Methods

### 3.1. Sample Preparation

Galβ1-4Glcβ-MP (**1**) was purchased from Tokyo Chemical Industry Co. and used without any further purification for ESI-MS measurement. The preparation of glycan **2** is shown in Scheme 1. The preparation of glycan **3a** and glycan **3b** is shown in Schemes S1 and S2. These synthetic glycans were purified by reversed-phase chromatography and characterized by NMR (Bruker AVANCE NEO 400, Bruker, Billerica, MA, USA) and MALDI-TOF-MS (Bruker microflex) measurements. High-resolution MS (HRMS) data were recorded using a Bruker scimaX mass spectrometer. PD MiniTrap G-10 columns (Cytiva, Marlborough, MA, USA) were used to desalt the synthetic samples prior to ESI–IM–MS measurements. 

### 3.2. ESI–IM–MS Measurement

Each glycan (0.1 mg/mL) dissolved in distilled water or methanol (HLC-SOL, KANTO Chemical Co., Tokyo, Japan) was used for ESI–IM–MS measurements. All ESI-MS and ESI–IM–MS measurements were performed using Waters SYNAPT XS (Waters Co., Milford, MA, USA). The ion source and desolvation gas temperatures were set at 35 °C unless otherwise noted. The other ion source conditions were as follows: capillary voltage, 1.0 kV; sampling cone voltage, 60 V; and desolvation gas flow, 800 L/h. For ion mobility analysis, nitrogen gas was used as a drift gas, and the wave velocity and wave height were set at 1100 m/s and 40 V, respectively. All ESI–IM–MS measurements were performed three times.

### 3.3. Synthesis of Trisaccharides

Reagents and solvents were purchased from Tokyo Chemical Industry Co. (Tokyo, Japan) and KANTO Chemical Co. and used without any further purification. Column chromatography was performed using a Biotage Isolera medium-pressure liquid chromatography system with Sfär Silica HC D columns (Biotage, Uppsala, Sweden). 

Monosaccharide derivative **4** (2.68 mmol) and disaccharide derivative **5** (1.34 mmol) were dissolved in dichloromethane dehydrated (22 mL). Activated molecular sieves 3A was added into the flask and the mixture was stirred at −60 °C. Trimethylsilyl trifluoromethanesulfonate (0.27 mmol) was added into the flask and the mixture was stirred for 12 h at −60 °C under a nitrogen atmosphere. When the completion of the reaction was checked by TLC, triethylamine was added for neutralization. After filtration and subsequent concentration, the mixture was purified by column chromatography (hexane/ethyl acetate) and column chromatography (toluene/ ethyl acetate) to give **6** (0.33 mmol, yield of 25%).

Trisaccharide derivative **6** (0.33 mmol) was dissolved in methanol (10 mL), and palladium hydroxide on carbon (3.3 mmol) was added into the flask and stirred for 12 h at 25 °C under a hydrogen atmosphere. When the completion of the reaction was checked by TLC, the solid was filtered off and the solvent was concentrated. The residue was dissolved in pyridine (5 mL), and acetic anhydride (2.4 mmol) was added into the flask at 0 °C. 4-Dimethylaminopyridine (0.23 mmol) was added into the flask and the mixture was stirred for 12 h at 25 °C under a nitrogen atmosphere. When the completion of the reaction was checked by TLC, the reaction mixture was concentrated. Ethyl acetate was added and the organic layer was washed sequentially with 0.1 M aqueous HCl, saturated aqueous NaHCO_3_, and brine. The organic layer was concentrated and the crude residue was purified by column chromatography (hexane/ethyl acetate) to give **7** (0.22 mmol, yield of 67% in two steps).

Trisaccharide derivative **7** (0.22 mmol) was dissolved in acetonitrile (5 mL) and toluene (7 mL) and cooled to −15 °C. Ammonium cerium (IV) nitrate (1.03 mmol) dissolved in distilled water (0.5 mL) was slowly added into the flask and the mixture was stirred for 5 h at −15 °C. When the completion of the reaction was checked by TLC, the reaction mixture was concentrated. Ethyl acetate was added and the organic layer was washed with saturated aqueous NaHCO_3_ and brine. The organic layer was concentrated and the crude residue was purified by column chromatography (hexane/ethyl acetate) to give **8** (0.14 mmol, yield of 64%).

Compound **8** (0.14 mmol) was dissolved in dichloromethane dehydrated (5 mL) and cooled to 0 °C, and 1,8-diazabicyclo [5.4.0]-7-undecene (0.04 mmol) was added into the flask. Trichloroacetonitrile (0.85 mmol) was added into the flask and the mixture was stirred for 3 h at 0 °C under a nitrogen atmosphere. When the completion of the reaction was checked by TLC, the mixture was concentrated and purified by column chromatography (hexane/ethyl acetate) to give **9** (0.098 mmol, yield of 70%).

Glycan donor **9** (0.098 mmol) and 2-(benzyloxycarbonylamino)-1-ethanol (0.16 mmol) were dissolved in dehydrated dichloromethane (4 mL). Activated molecular sieves 3A was added into the flask and the mixture was stirred at 0 °C. Trimethylsilyl trifluoromethanesulfonate (0.03 mmol) was added into the flask and the mixture was stirred for 4 h at 0 °C under a nitrogen atmosphere. When the completion of the reaction was checked by TLC, the reaction mixture was concentrated. Ethyl acetate was added and the organic layer was washed with saturated aqueous NaHCO_3_ and brine. The organic layer was concentrated and the crude residue was purified by column chromatography (hexane/ethyl acetate) to give **10** (0.045 mmol, yield of 46%).

Compound **10** (0.045 mmol) was dissolved in methanol (3 mL), and palladium hydroxide on carbon (0.45 mmol) was added into the flask and stirred for 12 h at 25 °C under a hydrogen atmosphere. When the completion of the reaction was checked by TLC, the solid was filtered off and the solvent was concentrated. The residue was dissolved in methanol (3 mL), and potassium carbonate (0.3 mmol) was added into the flask and stirred for 12 h at 25 °C. When the completion of the reaction was checked by TLC, the reaction mixture was concentrated and purified on an ODS column to give glycan **3a** (0.018 mmol, yield of 40% in two steps). HRMS: *m*/*z* calcd for C_20_H_38_NO_16_: 548.21851 [M+H]^+^; found: 548.21820.

Monosaccharide derivative **11** (1.82 mmol) and disaccharide derivative **5** (1.51 mmol) were dissolved in dichloromethane dehydrated (10 mL). Activated molecular sieves 3A was added into the flask and the mixture was stirred at −40 °C. Trimethylsilyl trifluoromethanesulfonate (0.15 mmol) was added into the flask and the mixture was stirred for 12 h at −40 °C under a nitrogen atmosphere. When the completion of the reaction was checked by TLC, triethylamine was added for neutralization. After filtration and subsequent concentration, the mixture was purified by column chromatography (hexane/ethyl acetate) to give **12** (1.35 mmol, yield of 89%).

Trisaccharide derivative **12** (0.54 mmol) was dissolved in methanol (6 mL), palladium hydroxide on carbon (5.4 mmol) was added into the flask, and the mixture was stirred for 12 h at 25 °C under a hydrogen atmosphere. When the completion of the reaction was checked by TLC, the solid was filtered off and the solvent was concentrated. The residue was dissolved in pyridine (5 mL), and acetic anhydride (5.3 mmol) was added into the flask at 0 °C. 4-Dimethylaminopyridine (0.49 mmol) was added into the flask and the mixture was stirred for 12 h at 25 °C under a nitrogen atmosphere. When the completion of the reaction was checked by TLC, the reaction mixture was concentrated. Ethyl acetate was added and the organic layer was washed sequentially with 0.1 M aqueous HCl, saturated aqueous NaHCO₃, and brine. The organic layer was concentrated and the crude residue was purified by column chromatography (hexane/ethyl acetate) to give **13** (0.42 mmol, yield of 78% in two steps).

Trisaccharide derivative **13** (0.42 mmol) was dissolved in acetonitrile (10 mL) and toluene (5 mL) and stirred at −15 °C. Ammonium cerium (IV) nitrate (0.85 mmol) dissolved in distilled water (2.5 mL) was added dropwise into the flask and the mixture was stirred for 3 h at −15 °C. When the completion of the reaction was checked by TLC, the reaction mixture was concentrated. The crude residue was purified by column chromatography (hexane/ethyl acetate) to give **14** (0.22 mmol, yield of 53%).

Compound **14** (0.22 mmol) was dissolved in dehydrated dichloromethane (4 mL) and cooled to 0 °C. 1,8-Diazabicyclo[5.4.0]undec-7-ene (0.03 mmol) and trichloroacetonitrile (1.34 mmol) were added into the flask and the mixture was stirred for 3 h at 0 °C under a nitrogen atmosphere. 1,8-Diazabicyclo[5.4.0]undec-7-ene (0.15 mmol) and trichloroacetonitrile (0.66 mmol) were added and the reaction continued for 2 h at 0 °C. When the completion of the reaction was checked by TLC, the mixture was concentrated and purified by column chromatography (hexane/ethyl acetate) to give compound **15** (0.18 mmol, yield of 82%).

Glycan donor **15** (0.18 mmol) and 2-(benzyloxycarbonylamino)-1-ethanol (0.26 mmol) were dissolved in dichloromethane dehydrated (4 mL). Activated molecular sieves 3A was added into the flask and the mixture was stirred at 0 °C. Trimethylsilyl trifluoromethanesulfonate (0.04 mmol) was added into the flask and the mixture was stirred for 2 h at 0 °C under a nitrogen atmosphere. When the completion of the reaction was checked by TLC, the reaction mixture was filtered and concentrated. Ethyl acetate was added and the organic layer was washed with saturated aqueous NaHCO_3_ and brine. The organic layer was concentrated and the crude residue was purified by column chromatography (hexane/ethyl acetate) to give **16** (0.07 mmol, yield of 39%).

Compound **16** (0.06 mmol) was dissolved in methanol (2 mL), and palladium hydroxide on carbon (0.29 mmol) was added into the flask and stirred for 12 h at 25 °C under a hydrogen atmosphere. When the completion of the reaction was checked by TLC, the solid was filtered off and the solvent was concentrated. The residue was dissolved in methanol (2 mL), sodium methoxide (0.24 mmol) in methanol (2 mL) was added into the flask, and the mixture was stirred for 12 h at 25 °C. Sodium methoxide (0.24 mmol) was added and the reaction continued for 4 h at 25 °C. When the completion of the reaction was checked by TLC, the reaction mixture was concentrated and purified on an ODS column to give glycan **3b** (0.01 mmol, yield of 17% in two steps). HRMS: *m*/*z* calcd for C_20_H_38_NO_16_: 548.21851 [M+H]^+^; found: 548.21818.

## 4. Conclusions

In this study, IM–MS measurements of Galβ1-4Glcβ, Galα1-4Galβ1-4Glcβ, and Galβ1-4Galβ1-4Glcβ glycans were performed using the ethylamine tag as a protonation site. The introduction of the protonation tag provided mobility data that directly reflected the structural characteristics of the glycans. The monoprotonated glycan ions exhibited different mobility behavior compared with the glycans ionized with sodium adduct ions. The observed arrival time distribution of the monoprotonated ions showed the coexistence of multiple conformations of the glycans. However, the interaction with the sodium ion perturbed the glycan conformational dynamics. The effects of the ionization conditions on the results of mobility analysis of the glycan ions were also examined. Ionization at high temperatures and with organic solvents increased the conformational dynamics of the glycan ions and broadened their arrival time distributions. These findings highlight the potential application of ion mobility spectroscopy to the structural analysis of glycans.

## Data Availability

The original contributions presented in this study are included in the article. Further inquiries can be directed to the corresponding author(s).

## Supplementary Materials

The following supporting information can be downloaded at: https://www.mdpi.com/article/10.3390/molecules30102177/s1, Schemes S1 and S2: Preparation of glycan 3a and glycan 3b.
