# Supplementary material for: Effects of Different Adduct Ions, Ionization Temperatures, and Solvents on the Ion Mobility of Glycans
Source: Molecules. 2025 May 15;30(10):2177. doi: 10.3390/molecules30102177 (PMC12114495; doi:10.3390/molecules30102177)
Supplement: Supplementary file 1 [file molecules-30-02177-s001.zip › molecules-3635429-supplementary.pdf]

Supplementary Materials

Effects of Different Adduct Ions, Ionization Temperatures, and Solvents on the Ion Mobility of Glycans

Hao Feng and Takumi Yamaguchi*

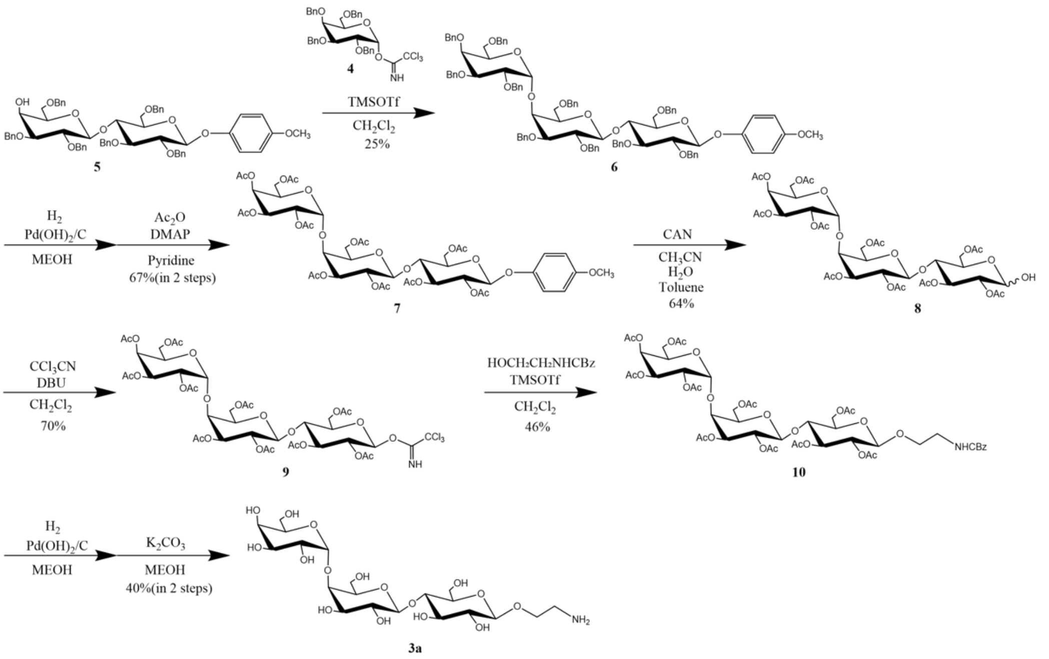

Scheme S1. Preparation of glycan **3a**.

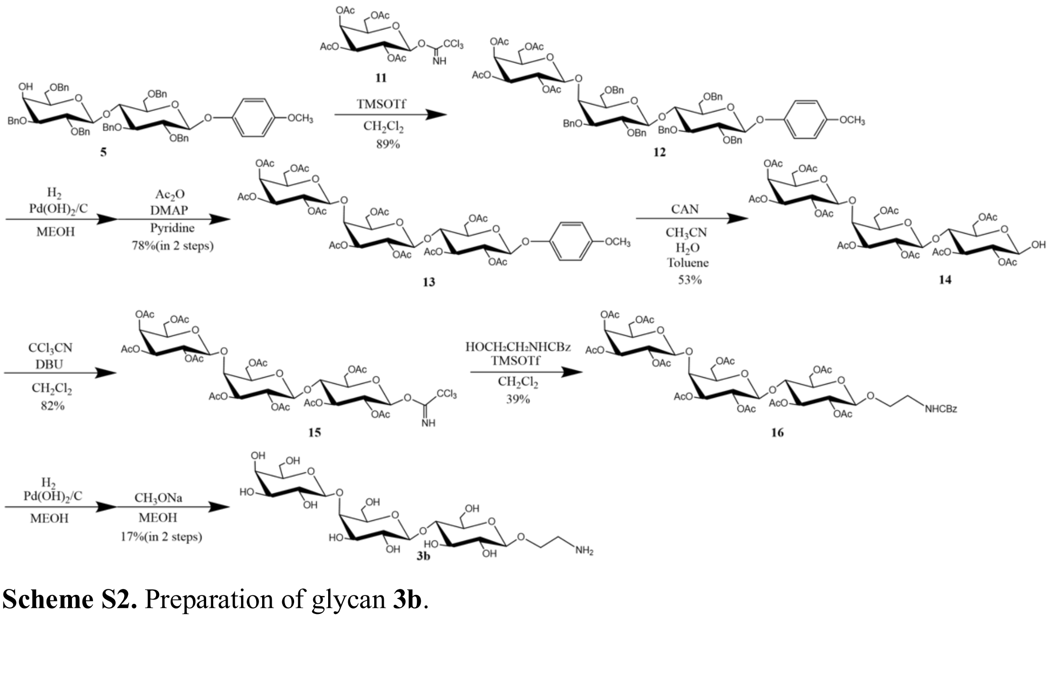

Scheme S2. Preparation of glycan **3b**.
